# High-mobility group box-1 and biomarkers of inflammation in the vitreous from patients with proliferative diabetic retinopathy

**Published:** 2011-07-06

**Authors:** Ahmed M. Abu El-Asrar, Mohd Imtiaz Nawaz, Dustan Kangave, Karel Geboes, Mohammad Shamsul Ola, Saif Ahmad, Mohamed Al-Shabrawey

**Affiliations:** 1 Department of Ophthalmology, College of Medicine, King Saud University, Riyadh, Saudi Arabia; 2 Laboratory of Histochemistry and Cytochemistry, University of Leuven, Belgium; 3 Department of Oral Biology, Ophthalmology, College of Dental Medicine and College of Medicine, Georgia Health Sciences University (GHSU), Augusta, GA

## Abstract

**Purpose:**

To measure levels of high-mobility group box −1 (HMGB1) and soluble receptor for advanced glycation end products (sRAGE) in the vitreous fluid from patients with proliferative diabetic retinopathy (PDR) and to correlate their levels with clinical disease activity and the levels of the inflammatory biomarkers monocyte chemoattractant protein-1 (MCP-1), soluble intercellular adhesion molecule-1 (sICAM-1), interleukin-1β (IL-1β), and granulocyte macrophage colony-stimulating factor (GM-CSF). In addition, we examined the expression of HMGB1 in the retinas of diabetic mice.

**Methods:**

Vitreous samples from 29 PDR and 17 nondiabetic patients were studied by enzyme-linked immunosorbent assay. Retinas of mice were examined by immunofluorescence analysis and western blotting.

**Results:**

HMGB1 was detected in all vitreous samples and sRAGE was detected in 5 PDR samples. IL-1β was detected in 3PDR samples and GM-CSF was not detected. Mean HMGB1 levels in PDR with active neovascularization were twofold and threefold higher than that in inactive PDR and nondiabetic patients, respectively. Mean HMGB1 levels in PDR patients with hemorrhage were significantly higher than those in PDR patients without hemorrhage and nondiabetic patients (p=0.0111). There were significant correlations between levels of HMGB1 and levels of MCP-1 (r=0.333, p=0.025) and sICAM-1 (r=0.548, p<0.001). HMGB1 expression was also upregulated in the retinas of diabetic mice.

**Conclusions:**

Subclinical chronic inflammation might contribute to the progression of PDR.

## Introduction

Proliferative diabetic retinopathy (PDR) is a serious complication of diabetes mellitus and is characterized by epiretinal outgrowth of fibrovascular membranes at the vitreoretinal interface. Formation of fibrovascular tissue often leads to severe visual loss due to vitreous hemorrhage and/or tractional retinal detachment. Strong evidence indicates that chronic low-grade inflammation is implicated in the pathogenesis of diabetic retinopathy. Diabetic retinal vascular leakage, capillary nonperfusion, and endothelial cell damage are associated with leukocyte recruitment and adhesion to the retinal vasculature, findings that correlate with the increased expression of retinal intercellular adhesion molecule-1 (ICAM-1) and the leukocyte integrin CD18. Inhibition of ICAM-1 activity in animals deficient in the gene encoding for ICAM-1 or by a neutralizing antibody suppresses both retinal leukostasis and vascular leakage [1,2]. The causal relationship between inflammation and angiogenesis is now widely accepted [3]. An emerging issue in diabetic retinopathy research is the focus on the mechanistic link between activation of subclinical inflammation and angiogenesis. Several studies reported elevated levels of biomarkers of inflammation and endothelial dysfunction, including monocyte chemoattractant protein-1 (MCP-1), interferon-γ-inducible protein of 10 kDa, interleukin-6 (IL-6), tumor necrosis factor-α, soluble intercellular adhesion molecule-1 (sICAM-1), and soluble vascular cell adhesion molecule-1 (sVCAM-1) in the vitreous from patients with PDR and diabetic macular edema [4-8].

High-mobility group box −1 protein (HMGB1) is a nonhistone, DNA-binding, nuclear protein that is highly conserved during evolution. It stabilizes nucleosome formation and facilitates gene transcription. Necrotic cell death can result in passive leakage of HMGB1 from the cell as the protein is then no longer bound to DNA. In addition, HMGB1 can be actively secreted by different cell types, including activated monocytes and macrophages, mature dendritic cells, natural killer cells, and endothelial cells. Extracellular HMGB1 functions as a proinflammatory cytokine [3,9-11] and exhibits angiogenic effects [12-15]. HMGB1 signals through the receptor for advanced glycation end products (RAGE), a member of the immunoglobulin superfamily of receptors, leading to activation of the transcription factor nuclear factor κB (NF-κB), and induces the expression of various leukocyte adhesion molecules and pro-inflammatory cytokines and chemokines [3,9-11]. Several studies demonstrated that RAGE mediates the inflammatory [9-11] and angiogenic [12,16] activities of HMGB1.

Soluble RAGE (sRAGE), a truncated form of the receptor, is composed of the extracellular ligand-binding domain lacking the cytosolic and transmembrane domains (i.e., the part that transfers the signal to the cell). This soluble form of the receptor has the same ligand-binding capacity and competes with cell-bound RAGE for ligand binding, therefore functioning as a “decoy” abrogating cellular activation since the cell-surface receptor remains unoccupied. Indeed, it has been demonstrated in several experimental animal models that treatment with sRAGE prevents cell-bound RAGE signaling [17].

The aim of this study was to measure the levels of HMGB1 and sRAGE in the vitreous fluid from patients with PDR and to correlate their levels with clinical disease activity and the levels of other biomarkers of inflammation and endothelial dysfunction, including MCP-1, sICAM-1, IL-1β, and granulocyte macrophage colony-stimulating factor (GM-CSF). In addition, we investigated the expression of HMGB1 in the retinas of diabetic mice.

## Methods

### Vitreous samples

Undiluted vitreous fluid samples (0.3–0.6 ml) were obtained from 29 patients with PDR during pars plana vitrectomy. All surgeries were performed at King Abdulaziz University Hospital, Riyadh, Saudi Arabia. The indications for vitrectomy were traction retinal detachment and/or nonclearing vitreous hemorrhage. The clinical ocular findings were graded at the time of vitrectomy for the presence or absence of visible new vessels on the retina or optic disc. Patients with active PDR were graded as such on the basis of visible patent new vessels on the retina or optic disc. Their absence indicated involuted (inactive) PDR. Active PDR was present in 15 patients, and inactive PDR was present in 14 patients. Traction retinal detachment was present in 12 patients and vitreous hemorrhage in 20 patients. The diabetic patients were 24 males and five females, whose ages ranged from 30 to 70 years with a mean of 46.9±9.9 years. The duration of diabetes ranged from 7 to 32 years with a mean of 16.4±5.6 years. Twenty-three patients had insulin-dependent diabetes mellitus, and six patients had noninsulin-dependent diabetes mellitus. At presentation, the fasting blood glucose was uncontrolled in 24 patients and controlled in five patients. Twenty patients were receiving treatment for hypertension, and two patients had diabetic neophropathy. Lipid profile assays showed elevated serum levels in nine patients. The control group consisted of 17 patients who had undergone vitrectomy for the treatment of rhegmatogenous retinal detachment (RD) with no proliferative vitreoretinopathy. Controls were free from systemic disease. Vitreous samples were collected undiluted by manual suction to a syringe through the aspiratin line of vitrectomy, before opening the infusion line. The samples were centrifuged (5,000× g for 10 min, 4 °C), and the supernatants were aliquoted and frozen at −80 °C until assay. The study was conducted according to the tenets of the Declaration of Helsinki, and informed consent was obtained from all patients. The study was approved by the Research Center, College of Medicine, King Saud University.

### Animals

All procedures with animals were performed in accordance with the ARVO Statement for the Use of Animals in Ophthalmic and Vision Research and were approved by the institutional Animal Care and Use Committee, Georgia Health Sciences University (GHSU). C57Bl/6J mice (The Jackson Laboratory, Bar Harbor, ME), weighing 25 to 30 g at the beginning of the study, were used. Diabetes was induced by intravenous injection of streptozotocin (STZ; Sigma-Aldrich, St. Louis, MO; 55 mg/kg, dissolved in 0.1 M sodium citrate buffer [pH 4.5]). Mice were considered diabetic if their blood glucose was greater than 250 mg/dl. After 8 weeks the animals were sacrificed by carbon dioxide (CO_2_) inhalation and the retinas of one eye of each mouse were removed, snap frozen in liquid nitrogen, and stored at −80 °C to be analyzed by western blotting. The other eye was enucleated and embedded in optimal cutting temperature compound (Electron Microscopy Sciences, Hatfield, PA) to prepare frozen sections (10 μm).

### Enzyme-linked immunosorbent assay kits

Enzyme-linked immunosorbent assay (ELISA) kits for human sRAGE (Quantikine Human Soluble Receptor for Advance Glycation end Product, Cat No: DRG00; R&D systems, Minneapolis, MN), human GM-CSF (Quantikine Human Granulocyte Macrophage Colony Stimulating factor, Cat No: DGM00), human IL-1β (Quantikine Human Interleukin 1 beta, Cat No: DLB50), human MCP-1 (Quantikine Human Monocyte Chemotactic Protein 1, Cat No: SCP00), and human sICAM (Quantikine Human soluble Intercellular Adhesion Molecules, Cat No: DCD540) were purchased from R&D Systems. An ELISA kit for HMGB-1 was purchased from IBL International GMBH (Hamburg, Germany). The detection limit for each ELISA kit for sRAGE, GM-CSF, IL-1β, MCP-1, sICAM-1, and HMGB-1 is 4.12 pg/ml, 3 pg/ml, 1 pg/ml, 5 pg/ml, 96 pg/ml, and 200 pg/ml, respectively. The ELISA plate readings were done using FLUOstar Omega-Miroplate reader from BMG Labtech (Offenburg, Germany). Preparation of buffers and reagent was done using ultrapure deionized water from Milli-Q Integral 10 system, Millipore (Billerica, MA).

### Preparation of standards

To prepare the series of standards, we followed the serial dilution methods. For each ELISA kit, the undiluted standard serves as the highest standard and calibrator diluents serve as the zero standard. Depending upon the detection range for each ELISA kit, the supernatant vitreous obtained was used either directly or diluted with calibrator diluents supplied with the ELISA kit.

### Measurement of human soluble receptor for advanced glycation end products, granulocyte macrophage colony-stimulating factor, interleukin-1β, and monocyte chemoattractant protein-1

Quantification of human sRAGE, GM-CSF, IL-1β, and MCP-1 in the vitreous fluid was determined using ELISA kits according to the manufacturer’s instruction (R&D Systems). For the measurement of sRAGE and GM-CSF in vitreous, an amount of 100 µl of assay diluent RD1–60 and RD1–6 (R&D Systems), respectively, and 50 µl of either the standard controls for sRAGE and 100 µl standard control for GM-CSF or an undiluted vitreous ﬂuid sample for sRAGE and GM-CSF were added to each well of the ELISA plate. Similarly for the measurement of IL-1β and MCP-1 in vitreous, an amount of 50 µl of assay diluent RD1–83 (R&D Systems) and 200 µl of either the standard controls for IL-1β and MCP-1 or twofold-diluted vitreous for IL-1β and 20-fold-diluted vitreous for MCP-1 were added to each well of the ELISA plate. The assay was performed in duplicate for each standard or vitreous sample. At room temperature, the ELISA plate was incubated on a shaker for 2 h and was washed as per the kit instruction, using a 1× diluted washing solution (R&D Systems). An amount of 200 µl of polyclonal antibody against sRAGE, GM-CSF, IL-1β, and MCP-1 conjugated to horseradish peroxidase (R&D Systems) was added to each well of the ELISA plate, incubated for 2 h on the shaker at room temperature, and washed using the 1× diluted washing solution. Immediately after washing, a 200-µl substrate solution, prepared using a 1:1 ratio of color reagent A (hydrogen peroxide; R&D Systems) and color reagent B (tetramethylbenzidine as a chromogen; R&D Systems), was added to each well and kept for another 30 min of incubation at room temperature and protected from light. The reaction was completed with the addition of 50 µl of stop solution (2 N sulfuric acid; R&D Systems) to each well of the ELISA plate in the same order as the substrate solution had been added, and the optical density (OD) was immediately read at 450 nm, using a microplate reader with a wavelength correction of 540 nm. Using the four parameter fit logistic (4-PL) curve equation for making the standards curve, the actual concentration for each sample was calculated. For the vitreous fluid that had been diluted, the correction read from the standard curve obtained using 4-PL was multiplied by the dilution factors to get the actual reading for each sample.

### Measurement of human soluble intercellular adhesion molecule-1

The quantification of human sICAM in the vitreous fluid was determined using ELISA kits according to the manufacturer’s instruction (R&D Systems). First an amount of 100 µl of monoclonal antibody against sICAM conjugated to horseradish peroxidase was added to each well, followed by the immediate addition of 100 µl of either the standard controls or threefold diluted vitreous fluid sample to each well of the ELISA plate. The assay was performed in duplicate for each standard or vitreous sample. At room temperature, the ELISA plate was incubated on a shaker for 1.5 h and was washed four times using the 1× diluted washing solution (R&D Systems). Using the 4-PL curve equation for making the standards curve, the actual concentration for each sample was calculated. For calculating the actual reading for each sample, the correction read from the standard curve obtained using 4-PL was multiplied by the dilution factors (3 times). For measuring sICAM, we diluted the vitreous fluid 3 times with diluent buffer that was supplied with the kit. To get the actual concentration of sICAM we multiplied the calculated reading with the dilution factor 3.

### Measurement of human high-mobility group box−1

With little modification, the quantification of human HMGB1 in the vitreous fluid was determined using ELISA kits according to the manufacturer’s instruction (IBL International GMBH). For measuring within the high sensitivity range, 65 µl of diluents buffer (Dilbuf, IBL International) was added to each microtiter plate followed, by the addition of 35 µl of standard, positive control, and vitreous fluid in the respective microtiter plate. After a brief shaking of the plate for 30 s, the plate was covered with adhesive foil and incubated for 22 h at 37 °C. Using the wash solution (Hamburg, Germany) diluted 5 times with deionized water, each well was washed 5 times, and 100 µl polyclonal HMGB1 antibody conjugated to horseradish peroxidase was added to each well, which was then incubated for another 2 h at room temperature. After washing five times, 100 µl of substrate solution (color reagent A, tetramethylbenzidine and color reagent B, 0.005 M hydrogen peroxide) was added to each well (prepared 5 min before its addition), and wells were then kept for 30 min at room temperature. The reaction was completed by the addition of 100 µl of stop solution (0.35 M H_2_SO_4_), and the OD was measured at 450 nm using a microplate reader with a wavelength correction of 620 nm. Using the 4-PL curve for making the standards curve, the actual concentration was calculated for each sample and the controls.

### Immunofluorescence analysis

The retinal sections were fixed using 4% formalin, blocked with serum-free protein blocker (Dako, Carpinteria, CA) at 25 °C in Coplin jars for 1 h, and then incubated with primary antibody over night at 4 °C. We performed double labeling for rabbit polyclonal anti-HMGB1 (1:100; Abcam, Cambridge, MA) and isolectin B4 (Vector Laboratories, Burlingame, CA) as the vascular marker (15 μg/ml). After overnight incubation we washed the slides three times with PBS (1x Phosphate buffer saline, Cat. No. 70011, Invitrogen, New York, NY)-Triton X-100 (0.2%) and then used Oregon Green antirabbit IgG (Molecular Probes; Invitrogen Corporation, Carlsbad, CA) and Texas Red Avidin D (Vector Laboratories) for HMGB1 and isolectin labeling, respectively. Sections were rinsed and washed three times (5 min each)and then mounted on slides by using Vectashield mounting medium with 4’,6-diamidino-2-phenylindole (DAPI) as a nuclear marker (Vector Laboratories), and examined by confocal microscopy (Carl Ziess NTS, LLC, Peabody, MA).

### Western-blot analysis

Retinas were homogenized in a modified radioimmunoprecipitation lysis (RIPA) buffer (20 mM Tris-HCl; pH 7.4), 2.5 mM EDTA, 50 mM NaF, 10 mM Na_4_P_2_O_7_, 1% Triton X-100, 0.1% sodium dodecyl sulfate [SDS], 1% sodium deoxycholate, and 1 mM phenylmethyl sulfonyl fluoride). The lysate was centrifuged at 14,000× g for 10 min at 4 °C, and the supernatant was collected. Equal amounts of protein were separated on 10% SDS-polyacrylamide gels. Proteins were transferred onto polyvinylidene difluoride membranes and blocked with 5% nonfat dry milk in Tris-buffered saline with 0.1% Tween-20 (TBS-T) for 1 h at room temperature. The membranes were incubated overnight with rabbit polyclonal anti-HMGB1 (1:1,000; Abcam, Cambridge, MA). After overnight incubation with primary antibody, membranes were washed three times with TBS-T (10 min each) and then incubated with antirabbit secondary horseradish peroxidase-conjugated antibody at room temperature for 1 h. Membranes were washed again three times with TBS-T (10 min each) and then the bands were visualized on a high-performance chemiluminescence film (GE Healthcare, Pittsburgh, PA) by using enhanced chemiluminescence plus (GE Healthcare) and quantified by densitometric analysis using Image processing and Analysis in Java (Image-J software, Open Source, Public Domain). As a control, the blots were stripped and detected with a mouse monoclonal anti-β-actin antibody (1:5,000; Santa Cruz Biotechnology, Inc., Santa Cruz, CA). All data from the three independent experiments were expressed as a ratio to OD.

### Statistical analysis

The Mann–Whitney test was used to compare means from two independent groups. Pearson correlation coefficients were computed to investigate correlations between variables. One-way ANOVA (ANOVA) and post-ANOVA pairwise comparisons of means were conducted using the Kruskal–Wallis test. For three groups, the critical Z value for post-ANOVA pairwise mean comparisons was Z=2.39 at a 5% level of significance. A p value less than 0.05 indicated statistical significance. Stepwise logistic regression analysis was conducted to identify the inflammatory biomarkers that had a strong relationship with the activity of PDR. Statistical Package for the Social Sciences Version 12 (SPSS 12.0), for Windows XP (SPSS Inc., Chicago, IL), and programs 3S and LR from Bio-Medical Data Processing Version 2007 (BMDP 2007) Statistical Software (Cork Technology Pack, Model Farm Road, Cord, Ireland) were used for the statistical analyses.

## Results

### Inflammatory biomarker levels in vitreous samples

HMGB1, MCP-1, and sICAM-1 were detected in all vitreous samples from patients with PDR and nondiabetic patients. sRAGE was detected in only five (17.2%) vitreous samples from patients with PDR at low levels (15.6, 45.8, 129.9, 29.5, and 69.1 pg/ml, respectively). None of the vitreous samples from nondiabetic patients showed detectable levels of sRAGE. IL-1β was detected in only three (10.3%) vitreous samples from patients with PDR at low levels (7.6, 5.9, and 6.3 pg/ml, respectively). None of the vitreous samples from nondiabetic patients showed detectable levels of IL-1β. GM-CSF was not detected in vitreous samples from patients with PDR or nondiabetic patients.

When patients with PDR were divided into those with active neovascularization and those with quiescent disease, the mean levels of HMGB1 in vitreous samples from patients with active PDR were approximately twofold higher than in inactive PDR patients and threefold higher than in nondiabetic patients. Likewise, the mean levels of MPC-1 and sICAM-1 were approximately twofold higher in active PDR patients than in inactive PDR patients (Table 1). Comparison of the mean levels of inflammatory biomarkers among active PDR patients, inactive PDR patients, and nondiabetic patients was conducted using the Kruskal–Wallis test, and the results are shown in Table 1. Mean levels differed significantly between the three groups for MCP-1 (p=0.007) and sICAM-1 (p<0.001) but not for HMGB1. Post-ANOVA pairwise comparisons of means indicated that mean MCP-1 levels were significantly higher in patients with active PDR than the mean levels in patients with inactive PDR (Z=2.91) and nondiabetic patients (Z=2.43). For sICAM-1, the mean levels for patients with active PDR and patients with inactive PDR were significantly higher than the mean levels in nondiabetic patients (Z=4.44, Z=2.93, respectively).

**Table 1:** Comparisons of mean inflammatory biomarker levels in relation to type of proliferative diabetic retinopathy (PDR).

**Disease group**	**HMGB1 (ng/ml)**	**MCP-1 (ng/ml)**	**sICAM-1 (ng/ml)**
Active PDR (n=15)	6.34±9.2	3.77±2.9	29.8±21.9
Inactive PDR (n=14)	3.06±3.1	1.93±1.5	15.45±5.6
Controls (n=17)	2.31±2.0	2.42±2.1	9.93±4.6
ANOVA p-value	0.257	0.007*	<0.001*

*Statistically significant at 5% level. Abbreviations: HMGB1 represents high-mobility group box −1; MCP-1 represents monocyte chemoattractant protein-1; sICAM-1 represents soluble intercellular adhesion molecule-1

When patients with PDR were divided into those with or without hemorrhage, the mean levels of inflammatory biomarkers differed significantly between PDR patients with hemorrhage, PDR patients without hemorrhage, and nondiabetic patients for HMGB1 (p=0.0111) and sICAM-1 (p<0.001) but not for MCP-1 (Table 2). Post-ANOVA pairwise comparisons of means highlighted that for HMGB1 the mean levels were significantly higher for PDR patients with hemorrhage than for PDR patients without hemorrhage (Z=2.82). For sICAM-1, the mean levels for PDR patients with or without hemorrhage were significantly higher than for nondiabetic patients (Z=4.42, Z=2.49, respectively).

**Table 2:** Comparisons of mean inflammatory biomarker levels in proliferative diabetic retinopathy (PDR) patients with or without hemorrhage

**Disease Group**	**HMGB1 (ng/ml)**	**MCP-1 (ng/ml)**	**sICAM-1 (ng/ml)**
PDR with hemorrhage (n=20)	6.18±8.1	2.91±1.9	25.53±19.3
PDR without hemorrhage (n=9)	1.60±1.8	2.83±3.6	17.05±11.8
Controls (n=17)	2.31±2.0	2.42±2.1	9.93±4.6
ANOVA p-value	0.0111*	0.1709	<0.001*

*Statistically significant at 5% level. Abbreviations: HMGB1 represents high-mobility group box −1; MCP-1 represents monocyte chemoattractant protein-1; sICAM-1 represents soluble intercellular adhesion molecule-1.

There were significant correlations between the vitreous fluid levels of HMGB1 and the levels of MCP-1 (r=0.333, p=0.025) and sICAM-1 (r=0.548, p<0.001). There was also a significant correlation between the vitreous fluid levels of MCP-1 and sICAM-1 (r=0.511, p<0.001) Stepwise logistic regression analysis was conducted to identify the inflammatory biomarkers that tended to relate most importantly to the activity of PDR and the presence of vitreous hemorrhage. High levels of sICAM-1 in the vitreous fluid were significantly associated with active neovascularization (odds ratio=1.14; 95% confidence interval [CI]=1.04–1.26). High levels of HMGB1 were associated with the presence of vitreous hemorrhage (odds ratio=6.55; 95% CI=0.616–69.6), but the association was not statistically significant.

When vitreous samples from patients with PDR were divided into those with or without detectable levels of sRAGE, mean levels of MCP-1 and sICAM-1 were significantly higher in vitreous samples with detectable levels of sRAGE compared to those without detectable levels of sRAGE (p<0.001 for both comparisons). The mean HMGB1 levels were higher in vitreous samples with detectable levels of sRAGE compared with those without detectable levels of sRAGE; the difference was marginally statistically significant (p=0.0532; Table 3).

**Table 3:** Comparisons of mean inflammatory biomarker levels in proliferative diabetic retinopathy vitreous samples with or without detectable soluble receptor for advanced glycation end products (sRAGE)

	**sRAGE detected**		
**Inflammatory biomarker**	**Yes**	**No**	**Mann–Whitney test p-value**
HMGB1 (ng/ml)	11.9±14.7	3.3±3.2	0.0532
MCP-1 (ng/ml)	6.9+3.8	2.0±0.9	<0.001*
sICAM-1 (ng/ml)	51.1±23.6	15.9±7.9	<0.001*

*Statistically significant at 5% level. Abbreviations: HMGB1 represents high-mobility group box −1; MCP-1 represents monocyte chemoattractant protein-1; sICAM-1 represents soluble intercellular adhesion molecule-1.

There were no significant relationships between vitreous levels of biomarkers of inflammation and systemic disease variables (Table 4).

**Table 4:** Relationship between inflammatory biomarker levels and systemic disease variables

**Variable**	**HMGB1 (ng/ml)**	**MCP-1 (ng/ml)**	**sICAM-1 (ng/ml)**
**Type of diabetes**			
Insulin-dependent	5.02±7.9	2.76±2.6	21.0±14.6
Non-insulin-dependent	3.04±2.0	3.17±2.1	19.5±8.2
p-value	0.834	0.294	0.816
**Fasting blood glucose**			
Controlled	2.92± 2.6	3.29±2.5	26.1±12.9
Uncontrolled	4.96±7.7	2.76±2.6	19.9±13.7
p-value	0.896	0.431	0.131
**Hyperlipidemia**			
Yes	3.51±2.2	4.34±4.2	30.3±13.7
No	6.04±10.4	2.78±2.3	19.9±15.7
p-value	0.708	0.399	0.092
**Hypertension**			
Yes	5.17±8.5	3.03±3.0	21.8±14.5
No	3.62±3.1	2.42±0.6	18.5±11.9
p-value	0.605	0.21	0.363

Abbreviations: HMGB1 represents high mobility group box−1; MCP-1 represents monocyte chemoattractant protein-1; sICAM-1 represents soluble intercellular adhesion molecule-1.

### Effect of diabetes on retinal expression of high-mobility group box −1 in experimental mice

**Figure 1:**
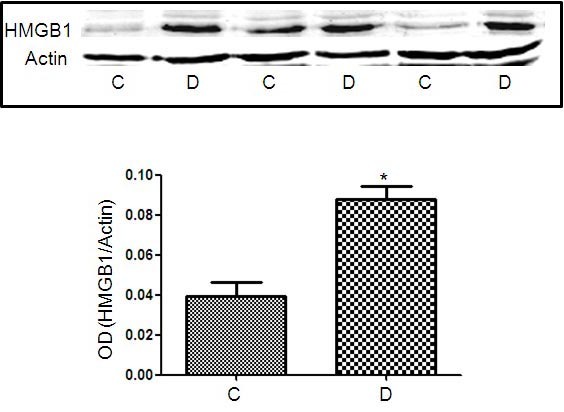
Western-blotting analysis of high-mobility group box −1 (HMGB1) in mouse retina. There is a significant increase in the expression of HMGB1 in the retinas of diabetic mice (D) compared to the nondiabetic control (C). OD is optical density

**Figure 2:**
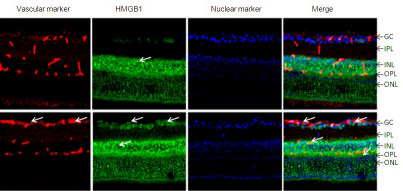
Immunolocalization of high-mobility group box −1 (HMGB1)  in control (upper row of panels) and diabetic (lower row of panels) retinas. Immunofluorescence using isolectin B4 as a vascular marker (red), a nuclear marker 4’,6-diamidino 2-phenylindole (DAPI), and HMGB1 antibody showed increased expression of HMGB1 in diabetic retina in comparison to the control. HMGB1 is localized mainly in ganglion cells (GC), inner nuclear layer (INL), and retinal vasculatures (arrows). Abbreviations: IPL represents inner plexiform layer; OPL represents outer plexiform layer; ONL represents outer nuclear layer.

Our previous studies reported early inflammatory response in retina of STZ-induced diabetic mice, characterized by increased leukocyte adhesion and vascular permeability [18]. We tested whether this early inflammatory response is associated with any change in the retinal levels of HMGB1. Our western blotting demonstrated a significant increase in HMGB1 expression in the retina of diabetic mice compared to the control (p=0.007; Figure 1). Using immunofluorescence we noticed that HMGB-1 is highly expressed in ganglion cells, inner nuclear layers, and retinal vasculatures (Figure 2).

## Discussion

In the present study we investigated the correlations between the levels of HMGB1 and sRAGE and the levels of other inflammatory biomarkers in vitreous samples from patients with PDR. In addition, we investigated the association of the inflammatory biomarkers in vitreous samples with the activity of PDR and the presence of vitreous hemorrhage. We also investigated the expression of HMGB1 in the retinas of diabetic mice. The main findings were as follows: (1) Vitreous levels of HMGB1, MCP-1, and sICAM-1 were higher in patients with active PDR compared with patients with inactive PDR and nondiabetic patients. (2) Vitreous levels of HMGB1 and sICAM-1 were higher in PDR patients with hemorrhage compared with PDR patients without hemorrhage and nondiabetic patients. (3) Among the inflammatory factors that we investigated, sICAM-1 had a stronger influence on the activity of PDR than the other factors. (4) There were significant correlations between the vitreous levels of HMGB1, MCP-1, and sICAM-1. (5) Levels of HMGB1, MCP-1, and sICAM-1 were higher in vitreous samples with detectable levels of sRAGE compared with vitreous samples without detectable levels of sRAGE. These findings suggest that HMGB1, MCP-1, and sICAM-1 play an important role in the progression of PDR. In addition, we demonstrated upregulation of HMGB1 expression in the retinas of diabetic mice.

In this study, HMGB1 and MCP-1 were detected in all vitreous samples from patients with rhegmatogenous retinal detachment (RD). Our results are consistent with previous reports that demonstrated elevated levels of HMGB1 and MCP-1 in the vitreous samples from patients with RD [5,19]. In vivo studies showed that induction of retinal detachment in rats induced upregulation of HMGB1 in both the photoreceptors and the other retinal cells and release of HMGB1 in the subretinal space [19]. These findings suggest that HMGB1 could be released not only in the subretinal space but also in the vitreous cavity after RD-induced photoreceptor degeneration. In the present study, PDR eyes with active neovascularization had a twofold increase in the vitreous level of HMGB1 when compared with those with quiescent disease and a threefold increase in the vitreous level of HMGB1 when compared with those with RD. These results are in agreement with a previous report in which we demonstrated that HMGB1 and RAGE were expressed by vascular endothelial cells and stromal cells in PDR fibrovascular epiretinal membranes and that there were significant correlations between the level of vascularization in PDR epiretinal membranes and the expression of HMGB1 and RAGE. Moreover, the expression of HMGB1 and RAGE in membranes from patients with active neovascularization was significantly higher than that in membranes from patients with inactive PDR [20]. In addition, we demonstrated that HMGB1 levels in vitreous samples of PDR patients with hemorrhage were significantly higher than in PDR patients without hemorrhage. Taken together, these findings suggest a role for the HMGB1/RAGE signaling axis in the progression of PDR. Recently, HMGB1 has been recognized as an angiogenic cytokine [12-15]. Several studies demonstrated that RAGE mediates the angiogenic activities of HMGB1 [12,16]. Another interesting role of HMGB1 in neovascularization is its ability to attract endothelial progenitor cells to sites of tissue injury and tumors to improve neovascularization in a RAGE-dependent manner [14]. In the present study we also demonstrated that HMGB1 expression was upregulated in the retinas of diabetic mice. This finding can be correlated with our previous report of increased ICAM-1 expression, leukocyte adhesion, and vascular permeability in retinas of diabetic mice [18]. Similarly, increased vascular [21] and renal [22] HMGB1 expression was recently demonstrated in diabetic animals. In addition, hyperglycemia-induced reactive oxygen species production increases the expression of HMGB1 and RAGE in endothelial cells [23]. Arimura et al. [19] showed that oxidative stress augmented HMGB1 in the nucleus and induced massive release of HMGB1 from cultured retinal cells to the cell supernatants.

Sustained pro-inflammatory responses in diabetic retinopathy are often associated with angiogenesis [1,2,4,24]. The causal relationship between inflammation and angiogenesis is now widely accepted [3]. The current data show a significant correlation between the vitreous levels of HMGB1 and that of the other inflammatory biomarkers MCP-1 and sICAM-1. Previous studies reported that HMGB1 activates human endothelial cells to upregulate the expression of RAGE and ICAM-1, to release MCP-1, and to increase neutrophil adhesion. This pro-inflammatory phenotype was mediated by the activation of NF-κB and was RAGE dependent as it was inhibited by antibodies directed toward RAGE [9-11].

Our results of increased levels of MCP-1 and sICAM-1 in the vitreous samples from patients with PDR are consistent with previous reports [4-6]. Furthermore, we found that MCP-1 and sICAM-1 levels in vitreous samples from active PDR cases were significantly higher than in inactive PDR cases. In a previous study we also demonstrated that MCP-1 protein was localized in vascular endothelial cells and myofibroblasts in PDR epiretinal membranes and that there was a significant correlation between the level of vascularization and the expression of MCP-1 in PDR epiretinal membranes [4]. Recently, MCP-1 and its regulation by high glucose levels in vascular cells has been implicated in the pathogenesis of the inflammatory process associated with diabetes [25]. In addition, MCP-1 has been recognized as an angiogenic chemokine [26,27]. In vivo angiogenesis assays showed that MCP-1-induced angiogenesis is as potent as that induced by vascular endothelial growth factor [26].

Elevated levels of sICAM-1 are thought to indicate a state of endothelial activation with consequent induction of immunological activity [28]. Several studies demonstrated elevated plasma levels of sICAM-1 and other inflammatory biomarkers and their association with microvascular complications in diabetic patients [29,30]. In addition, intensive therapy in patients with type 1 diabetes mellitus reduced serum levels of sICAM-1 [31]. In the current study, multivariate analysis demonstrated a significant association between active PDR and high levels of sICAM-1. In agreement with our findings, previous studies showed that vitreous levels of sICAM-1 were significantly associated with the severity of diabetic macular edema [7].

sRAGE, a truncated form of the receptor, binds ligands with affinity equal to that of cellular RAGE. It therefore has the ability to prevent RAGE signaling acting as a “decoy” by binding ligands and preventing them from reaching the cell-surface RAGE. In vitro, sRAGE added to cultured cells blocked the effects of RAGE ligands on expression of inflammatory markers, cellular migration and proliferation, and cytotoxicity [17]. sRAGE has successfully been used in a variety of animal disease models to antagonize RAGE-mediated pathologic processes [17,32]. Several studies showed that sRAGE might beneficially impact early vascular and neuronal dysfunction in the diabetic retina. Systemic administration of sRAGE inhibits blood–retinal barrier breakdown, leukostasis, and expression of ICAM-1 in the retina of diabetic animals [33]. In addition, attenuation of the RAGE axis with sRAGE ameliorated retinal neuronal dysfunction and reduced the development of capillary lesions in a murine model of nonproliferative diabetic retinopathy [34]. In the present study, the presence of detectable levels of sRAGE was associated with increased levels of HMGB1, MCP-1, and sICAM-1 in vitreous samples from patients with PDR. These results suggest that elevated levels of sRAGE in vitreous from patients with PDR potentially negatively regulates inflammation and that sRAGE is secreted extracellularly as a negative feedback mechanism to limit the inflammation. Similarly, in rheumatoid arthritis patients with erosive disease, there is a positive correlation between the white cell count and synovial sRAGE levels [35]. In addition, blood sRAGE levels are higher in systemic lupus erythematosus patients compared with controls. Compared with quiescent systemic lupus erythematosus, blood sRAGE levels are significantly increased during active disease [32]. It is, therefore, hypothesized that the association between detection of sRAGE and elevated levels of HMGB1 in vitreous samples from patients with PDR is protective against HMGB1-elicited cellular activation by complexing HMGB1 and promoting its clearance. On the other hand, in vitro studies revealed that treatment of cells with HMGB1 increased the formation of sRAGE released in the supernatants [32].

In addition to its role in mediating amplification of inflammation and angiogenesis, several studies demonstrated that extracellular HMGB1 can aggravate tissue damage in neuronal tissues after ischemia. HMGB1 is released to the extracellular space immediately after ischemic injury from neurons, and it subsequently induces neurodegeneration in the postischemic brain [36-38]. In these studies, extracellular HMGB1 plays a key role in the development of neuronal injury through microglial activation [38] and induction of apoptosis [39], excitatory amino acid release [40,41], and pro-inflammatory mediators [41].

In conclusion, our findings suggest the presence of a pro-inflammatory state in PDR as evidenced by increased levels of HMGB1, MCP-1, and sICAM-1 in the vitreous. Activation of subclinical chronic inflammation might contribute to the pathogenesis and progression of PDR. Moreover, HMGB1 expression was enhanced in the retinas of diabetic mice.
